# Causal relationships of circulating amino acids with cardiovascular disease: a trans-ancestry Mendelian randomization analysis

**DOI:** 10.1186/s12967-023-04580-y

**Published:** 2023-10-07

**Authors:** Song Hu, Zhennan Lin, Meng-Jin Hu, Jiang-Shan Tan, Ting-Ting Guo, Xin Huang, Lu Hua

**Affiliations:** 1 Key Laboratory of Pulmonary Vascular Medicine, State Key Laboratory of Cardiovascular Disease, Center for Respiratory and Pulmonary Vascular Diseases, Department of Cardiology, National Clinical Research Center of Cardiovascular Diseases, National Center for Cardiovascular Diseases, Fuwai Hospital, Chinese Academy of Medical Sciences and Peking Union Medical College, Beijing, 100037 China; 2 Fuwai Hospital, National Center for Cardiovascular Diseases, Chinese Academy of Medical Sciences and Peking Union Medical College, Beijing, 100037 China; 3 State Key Laboratory of Cardiovascular Disease, Fuwai Hospital, National Center for Cardiovascular Diseases, Chinese Academy of Medical Sciences and Peking Union Medical College, Beijing, 100037 China; 4 Department of Cardiology, The Second Medical Center & National Clinical Research Center for Geriatric Diseases, Chinese PLA General Hospital, Beijing, 100853 China; 5 Fuwai Hospital, Chinese Academy of Medical Sciences, Shenzhen, China

**Keywords:** Amino acid, Cardiovascular disease, Mendelian randomization, Causal relationship

## Abstract

**Background:**

Epidemiological studies demonstrated that multiple amino acids (AAs) were associated with cardiovascular diseases (CVDs), but whether these associations were causal remains unclear. This study aims to investigate the causal relationships between circulating levels of 20 AAs and 10 CVDs in European and East Asian populations by Mendelian randomization (MR).

**Methods:**

This MR study utilized single-nucleotide polymorphisms that were significantly associated with AAs as instrumental variables. Summary-level data for AAs and CVDs were obtained from public genome-wide association studies. The causal effects were primarily estimated by inverse variance weighting with multiplicative random effect method. Sensitivity analyses, including weighted median, weighted mode, and MR Egger regression, were used to test the robustness of our results.

**Results:**

In the European population, alanine and serine were inversely associated with angina pectoris (AP) and chronic heart failure, respectively. With each unit increase of leucine, the risk of ischemic stroke increased by 10%. Moreover, tyrosine was positively associated with AP and deep vein thrombosis. In the East Asian population, each unit increase in glycine was associated with 4.1% and 9.0% decreased risks of coronary artery disease (CAD) and myocardial infarction (MI), respectively. A unit increase in serine was associated with 13.1%, 12.6% and 15.5% decreased risks of AP, CAD and MI, respectively. Sensitivity analyses supported the robustness of our results.

**Conclusions:**

This MR study demonstrated significant causal effects of circulating levels of AAs on CVDs, indicating the potential use of AAs as biomarkers or as therapeutic targets for CVD in clinical scenarios.

**Graphical Abstract:**

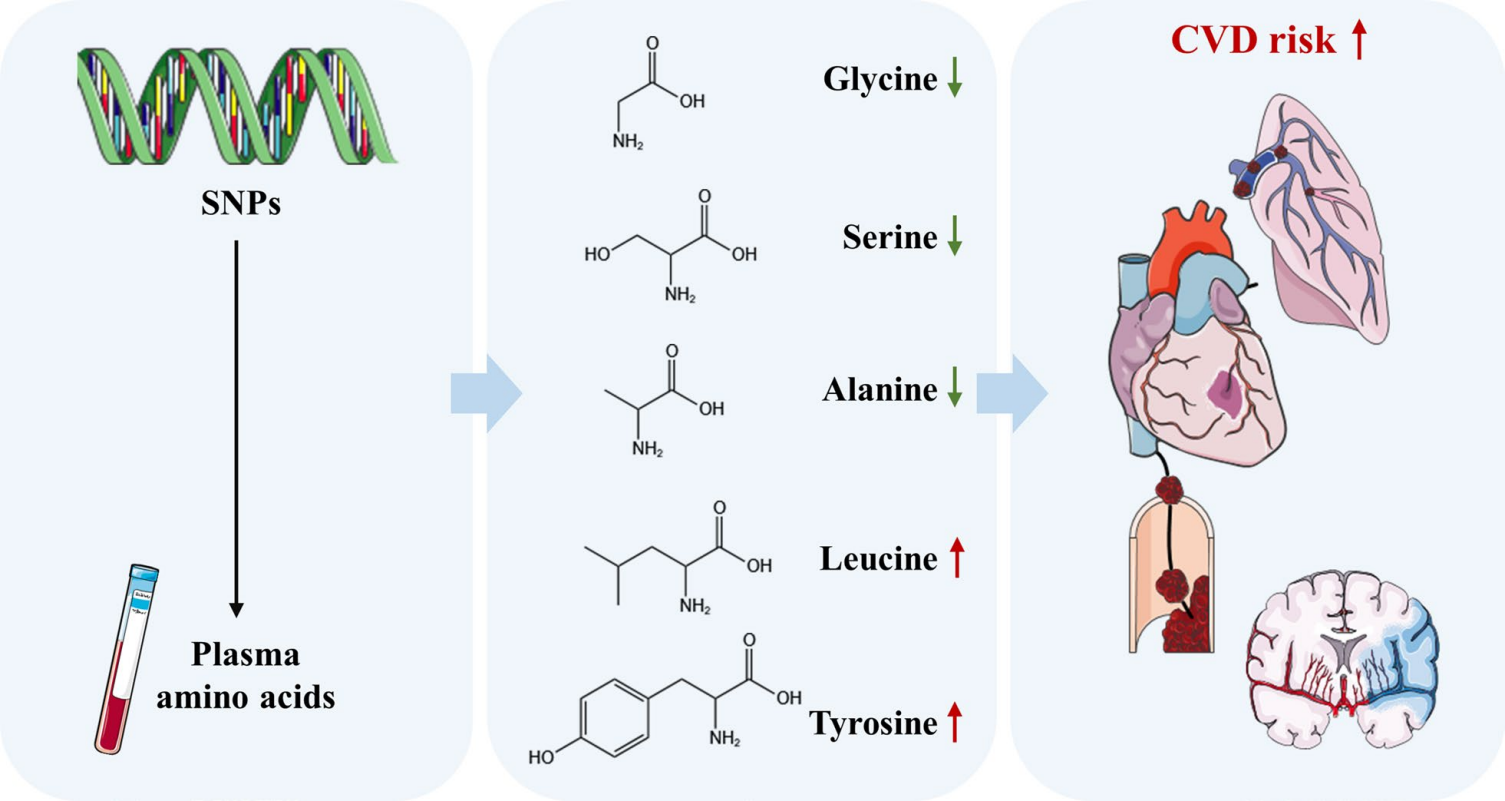

**Supplementary Information:**

The online version contains supplementary material available at 10.1186/s12967-023-04580-y.

## Background

Cardiovascular disease (CVD) is the leading cause of death worldwide [1]. Global Burden of Disease study estimated that nearly 523 million people were affected by CVD worldwide, and the number of deaths and years lived with disability due to CVD reached 18.6 million and 34.4 million, respectively [2]. With the growth of the aging population, the CVD burden is expected to increase, which necessitates more tasks for disease prevention. Over the past decades, researchers have established many risk factors for CVD, such as obesity, smoking, hyperlipidemia, hypertension and diabetes [3]. In addition to these conventional risk factors, there is an emerging recognition that metabolites such as circulating amino acids (AAs) are implicated in the development of CVD [4–6]. The potential roles of AAs, including as CVD biomarkers for disease prediction or as therapeutic targets for disease treatment, are promising in clinical scenarios.

Experimental evidences have shown that various AAs and their metabolism play important roles in regulating and maintaining vascular function, including vascular tone, coagulation and fibrinolysis, and immuno-inflammatory responses [7]. In some instances, the metabolism of AAs may also generate detrimental compounds that foster vascular disease [4, 5]. Observational studies have found that some AAs, such as arginine and tryptophan [8, 9], may be associated with a lower risk of CVD. Higher plasma levels of some other AAs, including branched chain amino acids (BCAAs, consisting of valine, leucine and isoleucine), tyrosine and phenylalanine [10, 11], predicted a higher CVD risk. Moreover, a recent large meta-analysis suggested that people with higher circulating isoleucine levels had higher CVD risk [12]. Although abundant evidence of the associations between AAs and CVD were provided, only a limited number of AAs were investigated, and whether these associations were causal remains unclear.

On account of the underlying reverse causality and confounding in observational studies, it is difficult to infer causality. Mendelian randomization (MR) is useful to investigate the causal effect of exposure on outcome [13, 14]. MR analysis uses genetic variants, commonly single-nucleotide polymorphisms (SNPs), as unbiased proxies for modifiable risk factors to test whether the risk factor is causally related to a disease. Given that the genetic variants are randomly allocated and determined at conception, MR study is analogous to a randomized controlled trial, and MR can overcome the limitations of observational studies.

To date, no MR studies have systematically examined the causal relationships of 20 genetically encoded AAs with CVD. In the present study, we performed a trans-ancestry MR study investigating the causal effects of 20 AAs on the risk of 10 CVDs in European population and East Asian (EAS) population, respectively. The results will provide insights regarding the potential role of circulating AAs in the prevention and treatment of CVD.

## Methods

### Study design

**Fig. 1:**
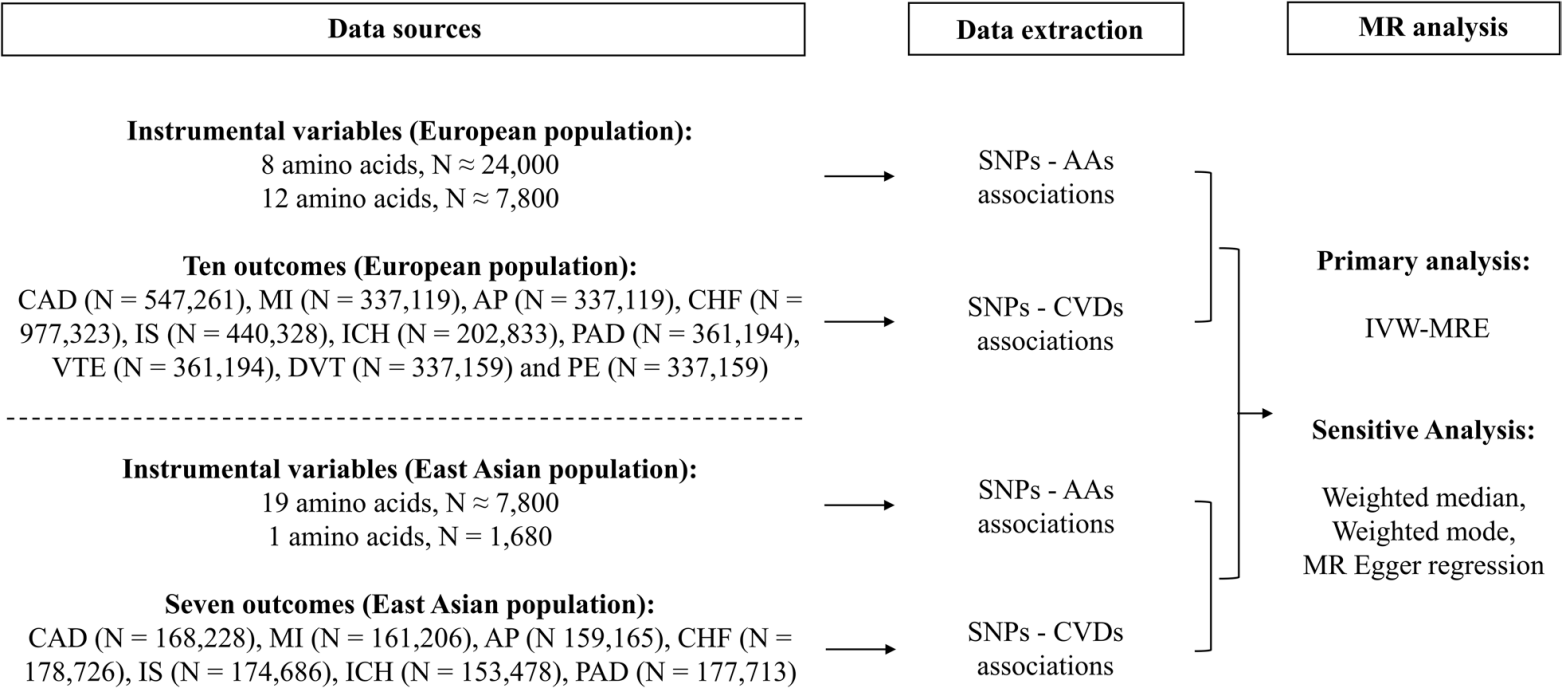
Overview of the study design. The flow chart showed the data collection and analysis for the current study. Details of the data sources can be found in Additional file 1: Table S1. We conducted a trans-ancestry MR study to evaluate the causalities between the circulating levels of 20 genetically encoded AAs and CVDs in European and East Asian populations, respectively. *AA* amino acid, *AP* angina pectoris, *CAD* coronary artery disease, *CHF* chronic heart failure, *CVD* cardiovascular disease, *DVT* deep vein thrombosis, *ICH* intracerebral hemorrhage, *IS* ischemic stroke, *IVW-MRE* inverse variance weighted with multiplicative random effects, *MI* myocardial infarction, *MR* Mendelian randomization, *PAD* peripheral arterial disease, *PE* pulmonary embolism, *SNP* single-nucleotide polymorphism, *VTE* venous thromboembolism

MR is an epidemiologic method that may be used to infer the causality of two variables, which should satisfy the following conditions: (1) the genetic variants that are used as instrumental variables (IVs) should be significantly associated with exposure; (2) the IVs are not associated with any confounders of exposure and outcome; (3) the IVs are conditionally associated with outcome through exposure. Based on the framework of two-sample MR (TSMR) analysis, we conducted a trans-ancestry MR study to evaluate the causalities between the circulating levels of 20 genetically encoded AAs and 10 CVDs in European population and EAS population, respectively. Nonoverlapping datasets were used for exposure and outcomes. The 10 CVDs included coronary artery disease (CAD), myocardial infarction (MI), angina pectoris (AP), chronic heart failure (CHF), ischemic stroke (IS), intracerebral hemorrhage (ICH), peripheral arterial disease (PAD), venous thromboembolism (VTE), deep vein thrombosis (DVT) and pulmonary embolism (PE). For EAS population, we included only seven CVDs due to the absence of genome-wide association study (GWAS) summary statistics of VTE, DVT, and PE. SNPs that were significantly associated with AAs were used as IVs. Summary-level data for all traits were obtained from published studies or the MRC integrative Epidemiology Unit (IEU) OpenGWAS data [15], which had been approved by their institutional review committees. An overview of the study design was shown in Fig. 1.

### Data source and instrumental variables

For the European population, two previous studies have conducted GWAS for AAs [16, 17], and we preferentially used summary data with larger sample size to select IVs. Summary statistics for alanine, glutamine, histidine, isoleucine, leucine, phenylalanine, tyrosine, and valine were from the study conducted in 2016 [16]. The GWAS for AAs in this study was meta-analyzed from 14 European cohorts containing approximately 24,000 individuals and 12.0 million SNPs, in which the plasma levels of AAs were quantified using high-throughput nuclear magnetic resonance (NMR) metabolomics platform and normalized before performing GWASs. Summary statistics for the other 12 AAs were from the study conducted in two European populations in 2014 [17], which contained approximately 7800 participants with 2.5 million SNPs. In this study, the plasma levels of AAs were profiled using an ultra-high performance liquid chromatography-tandem mass spectrometry (UPLC‒MS/MS) platform and log-transformed with base 10 before conducting GWASs. For the EAS population, we selected IVs of 19 AAs, except aspartic acid, based on the GWAS conducted in two Japanese cohorts (the TMM BirThree cohort study and the TMM CommCohort study), with approximately 7800 participants and 8.1 million SNPs [18]. In this study, AAs were measured using NMR and log-transformed and rank-based inverse normalized. Due to the absence of aspartic acid in the TMM study, we selected its IVs from a GWAS conducted in 1680 Chinese individuals containing 9.9 million SNPs [19]. Aspartic acid was profiled using UPLC‒MS/MS and log-transformed median-normalized before conducting GWAS.

Summary-level data of these 10 CVDs in the European population were obtained from a meta-GWAS of the UK Biobank (UKB) and the consortium of CARDIoGRAMplusC4D for CAD (N = 547,261) [20], the MRC IEU OpenGWAS data for MI (N = 337,119) and AP (N = 337,119) conducted in the UKB population [15], a meta-GWAS containing 26 studies for CHF (N = 977,323) [21], a meta-GWAS containing 17 European cohorts that was from the MEGASTROKE consortium for IS (N = 440,328) [22], the MRC IEU OpenGWAS data for ICH conducted in FinnGen population (N = 202,833), and the MRC IEU OpenGWAS data for PAD (N = 361,194), VTE (N = 361,194), DVT (N = 337,159) and PE (N = 337,159) based on the UKB population [15]. For the EAS population, the summary statistics of seven CVDs were obtained from the Biobank Japan (BBJ) project [18, 23]. Detailed information on the GWAS summary statistics was listed in Additional file 1: Table S1.

IVs of AAs were selected based on the following steps: (1) screening SNPs with *P* < 5 × 10^−8^; (2) using SNPs with F-statistic > 10 to avoid weak instrument bias; (3) identifying independent SNPs using linkage disequilibrium (LD) clumping with a r^2^ < 0.01 within 10,000 kbp window based on the ancestry-specific 1000 Genomes panel; (4) using Steiger filtering to remove reverse causal instruments; (5) removing potential pleiotropic SNPs by RadialMR [24].

### Statistical analysis

The causal effects of AAs on CVDs were evaluated based on the SNP-specific Wald ratio method, i.e., the effect sizes of IVs on outcomes divided by that on exposure. To ensure the stability of MR causal effects, each AA should contain at least three eligible IVs. We used the inverse variance weighting with multiplicative random effect (IVW-MRE) method to meta-analyzed Wald ratio estimates. The Bonferroni correction method was used to control type I error due to multiple comparisons, with *P* < 2.50 × 10^−4^ (0.05/20/10) for the European population and *P* < 3.57 × 10^−4^ (0.05/20/7) for the EAS population as the cut-offs of statistical significance. The causal effects of AAs on CVDs were reported as odds ratios (ORs) and 95% confidence intervals (CIs) per unit increase in AAs. Due to the different data processing for the original plasma levels of AAs in different studies, it was difficult to harmonize them. Therefore, the effect sizes of AAs on CVDs were only comparable among those AAs from the same study. Heterogeneity of the genetic variants was tested based on the Q statistics, and horizontal pleiotropy was assessed using the MR Egger intercept. *P* < 0.05 indicated existing heterogeneity or pleiotropy between IVs. In addition, to further control the potential horizontal pleiotropy, we also conducted sensitivity analysis using three robust methods: weighted median, weighted mode, and MR Egger regression. Those relationships with Bonferroni significance and directionally similar estimates across different methods were considered as strong evidence in support of causality, while those without Bonferroni significance but with *P* < 0.05 or with Bonferroni significance but without directionally similar estimates were considered suggestive evidence.

All analyses were performed using R version 4.0.5 (R Foundation for Statistical Computing, Austria), and the R packages “TwoSampleMR” and “RadialMR” were used for MR analyses.

## Results

**Fig. 2:**
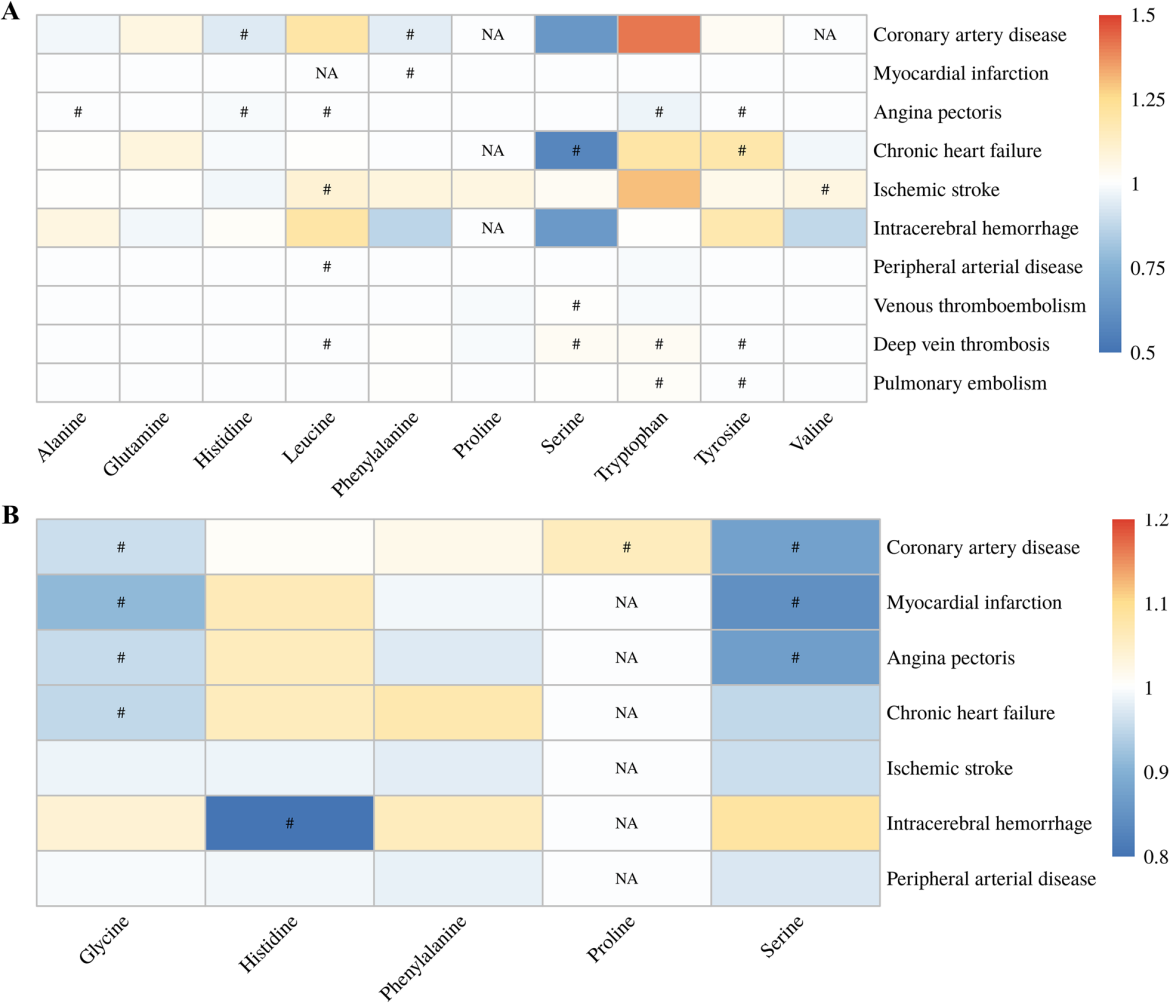
Relationships between AAs and CVDs among the European population (**A**) and EAS population (**B**). There were 10 AAs in the European population and five AAs in the EAS population that had sufficient eligible IVs (N_snp_ ≥ 3) to conduct MR analyses. ORs estimated using IVW-MRE were showed in the heatmap. ^#^*P* < 0.05; *NA* not available due to insufficient IVs, *AA* amino acid, *CVD* cardiovascular disease, *EAS* East Asian, *IV* instrumental variable, *IVW-MRE* inverse variance weighted with multiple random effect, *MR* Mendelian randomization, *OR* odds ratio, *SNP* single nucleotide polymorphism

There were 10 AAs in the European population and five AAs in the EAS population that had sufficient eligible IVs to conduct MR analyses, and the IV information was presented in Additional file 1: Table S2. For the European population, we identified 20 relationships that may be causal (*P* < 0.05), with eight AAs associated with at least one CVD, including alanine, histidine, leucine, phenylalanine, serine, tryptophan, tyrosine and valine (Fig. 2A). We observed that four AAs were associated with at least one CVD (*P* < 0.05) in the EAS population, including glycine, histidine, proline and serine, with nine potential causal relationships (Fig. 2B). It should be noted that we did not observe the same causal relationships between AAs and CVDs in European and EAS populations.

**Fig. 3:**
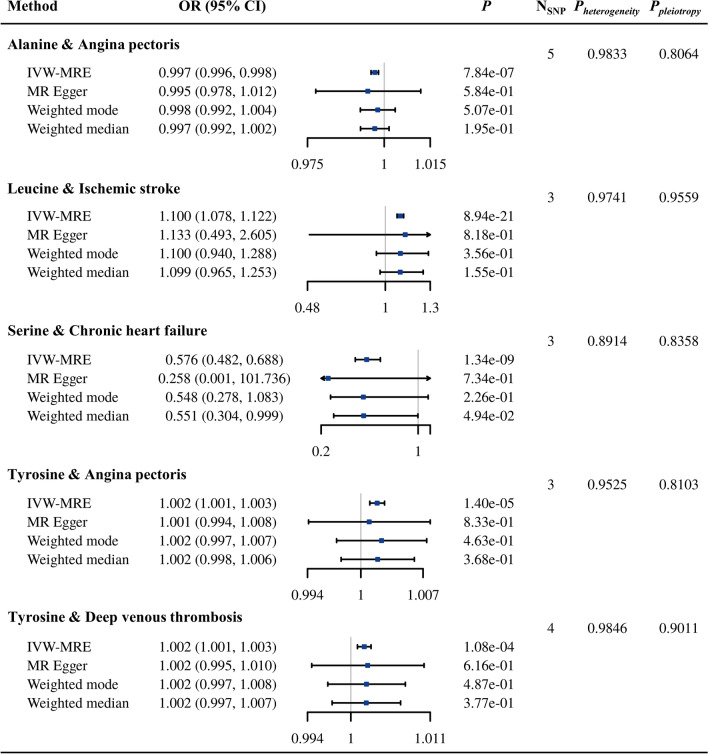
Causal relationships between AAs and CVDs with strong evidence in the European population. *AA* amino acid, *CVD* cardiovascular disease, *CI* confidence interval, *IVW-MRE* inverse variance weighted with multiple random effect, *MR* Mendelian randomization, *OR* odds ratio, *SNP* single nucleotide polymorphism

**Fig. 4:**
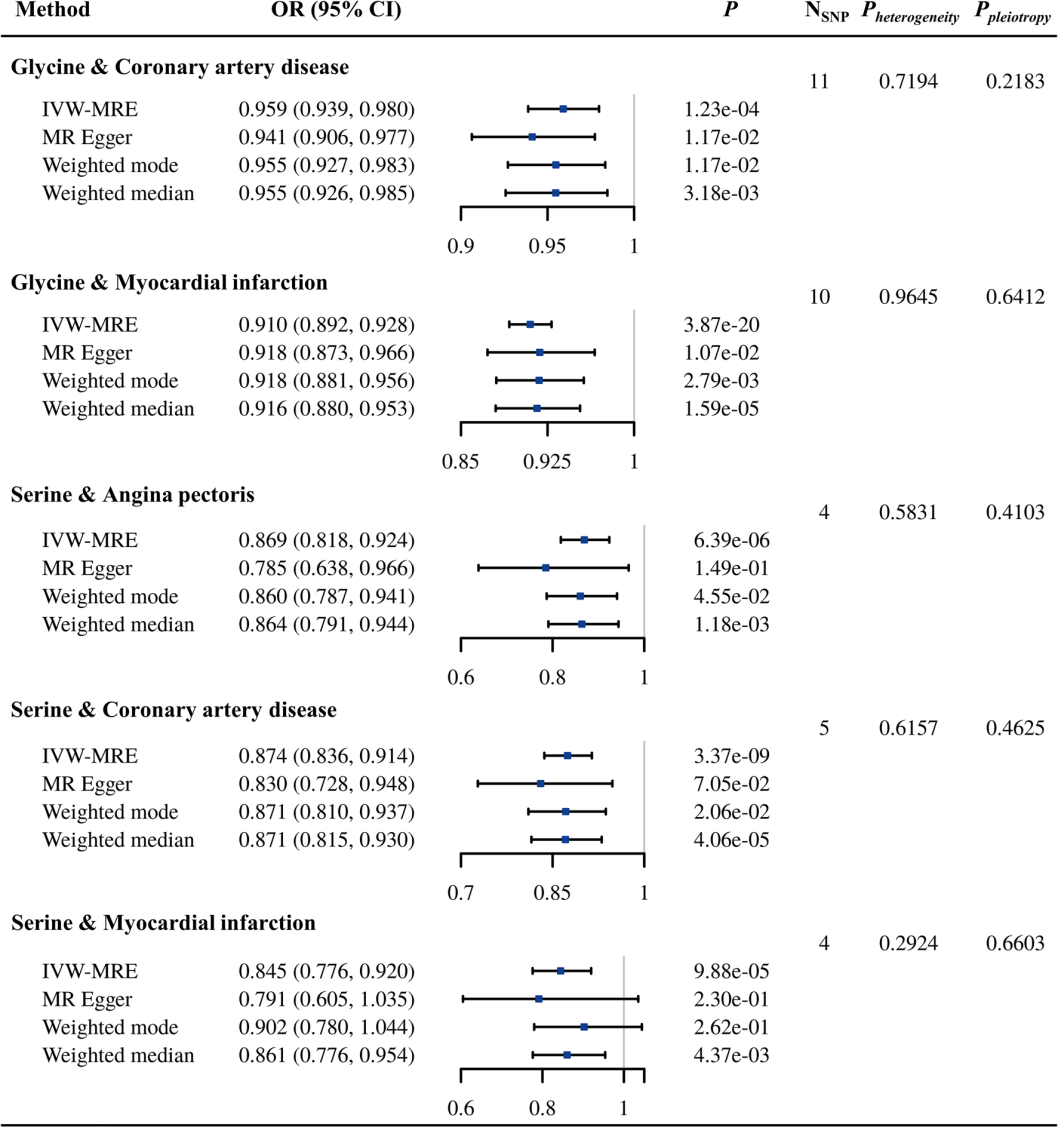
Causal relationships between AAs and CVDs with strong evidence in the EAS population. Due to the small number of eligible instrumental variables, there were inconsistencies between the estimates of CIs and P-values for several results. *AA* amino acid, *CVD* cardiovascular disease, *CI* confidence interval, *EAS* East Asian, *IVW-MRE* inverse variance weighted with multiple random effect, *MR* Mendelian randomization, *OR* odds ratio, *SNP* single nucleotide polymorphism

As demonstrated in Fig. 3, five causal relationships with strong evidence were identified for the European population in our study. Specifically, alanine and serine were inversely associated with AP (OR = 0.997, 95% CI 0.996–0.998, *P*_*IVW-MRE*_ = 7.84 × 10^−7^) and CHF (OR = 0.576, 95% CI 0.428–0.688, *P*_*IVW-MRE*_ = 1.34 × 10^−9^), respectively. With each one unit increase in leucine level, the risk of IS increased by 10% (OR = 1.100, 95% CI 1.078–1.122, *P*_*IVW-MRE*_ = 8.94 × 10^−21^). Moreover, the circulating tyrosine level was positively associated with the risk of AP (OR = 1.002, 95% CI 1.001–1.003, *P*_*IVW-MRE*_ = 1.40 × 10^−5^) and DVT (OR = 1.002, 95% CI 1.001–1.003, *P*_*IVW-MRE*_ = 1.08 × 10^−4^). For the EAS population, we also identified five relationships with strong evidence. All these relationships focused on the associations of glycine and serine with atherosclerotic diseases (CAD, MI and AP) (Fig. 4). Specifically, each one-unit increase in glycine was associated with 4.1% and 9.0% decreases in the risk of CAD (OR = 0.959, 95% CI 0.939–0.980, *P*_*IVW-MRE*_ = 1.23 × 10^−4^) and MI (OR = 0.910, 95% CI 0.892–0.928, *P*_*IVW-MRE*_ = 3.87 × 10^−20^), respectively. A one-unit increase in serine was associated with 13.1%, 12.6% and 15.5% decreases in AP (OR = 0.869, 95% CI 0.818–0.924, *P*_*IVW-MRE*_ = 6.39 × 10^−6^), CAD (OR = 0.874, 95% CI 0.836–0.914, *P*_*IVW-MRE*_ = 3.37 × 10^−9^) and MI (OR = 0.845, 95% CI 0.776–0.920, *P*_*IVW-MRE*_ = 9.88 × 10^−5^), respectively. There was no heterogeneity or horizontal pleiotropy among IVs for these relationships (*P* > 0.05), and all robust methods had directionally similar estimates.

In addition, we identified 15 and four relationships with suggestive evidence for the European and EAS populations, respectively (Additional file 1: Tables S3 and S4). For instance, histidine was inversely associated with CAD and AP in the European population and inversely associated with ICH in the EAS population. Moreover, in the European population, two BCAAs (leucine and valine) were identified to be associated with AP, PAD, IS, or DVT; three aromatic AAs (phenylalanine, tryptophan and tyrosine) were related to CAD, MI, AP, CHF, DVT or PE; and serine was positively associated with DVT and VTE. In the EAS population, proline was identified to be positively related to CAD, and glycine was inversely associated with AP and CHF.

## Discussion

The present MR analysis identified five relationships with strong evidence between AAs and CVDs in European and EAS populations, respectively. In the European population, higher circulating level of BCAA (leucine) was associated with an increased risk of IS, and higher circulating level of tyrosine was associated with an increased risk of AP or DVT. In addition, alanine and serine were inversely associated with AP and CHF, respectively. In the EAS population, the protective effects of serine and glycine on atherosclerotic diseases (including CAD, MI and AP) were observed. This study provided insight for the prevention or treatment of CVDs.

Many observational studies suggested that higher levels of circulating BCAAs were associated with higher CVD risk [10, 25, 26]. A case‒control study reported that circulating BCAAs were positively associated with the risk of CVD, especially stroke [10], and our study confirmed the causal relationship of leucine with the increased risk of IS in European population. In addition, some other positive relationships of BCAAs with CVDs were suggestive for European population as well, such as leucine with AP, PAD and DVT and valine with IS. One possible mechanism may be that elevated levels of BCAAs could induce proinflammatory and oxidative status in both circulating blood cells and endothelial cells, thereby resulting in inflammatory cell adhesion and endothelial dysfunction, which are involved in the pathophysiological process of CVDs [27, 28]. Moreover, a previous study demonstrated that BCAAs significantly enhanced platelet activity in human and promoted arterial thrombosis formation in mice, and the increased tropomodulin-3 propionylation mediated by BCAAs metabolic products played an essential role in this process [29]. These findings suggested that targeting BCAAs and their metabolism pathway was a promising approach for anti-thrombosis therapy. Although these explanations have been proposed, ongoing studies are warranted to clarify the exact role of BCAAs in CVDs.

In the European population, we identified that serine was inversely associated with CHF. Intriguingly, the five causal relationships with strong evidence in the EAS population were all focused on the inverse associations of glycine and serine with atherosclerotic diseases, including CAD, MI and AP. Protective effects of glycine on AP and CHF in the EAS population were also suggestive. Glycine and serine are two biochemically closely related AAs, and serine is the precursor for the synthesis of glycine. Both play an important role in neurological function, metabolic regulation and antioxidative reaction. Observational studies suggested that glycine and serine had antihypertensive effects [30–32]. Furthermore, one study showed that higher plasma glycine was associated with a decreased risk of CAD, and the inverse association was particularly strong in patients with apolipoprotein B, low-density lipoprotein cholesterol, or apolipoprotein A-1 levels above the median [33]. Our findings were in line with these studies and confirmed the causal relationships of glycine and serine with CVDs. Therefore, the cardioprotective effects of glycine and serine could be ascribed to their anti-inflammatory and antioxidant properties [34–36].

As a non-essential AA, tyrosine is a precursor for cell-specific syntheses of proteins, including thyroid hormone, dopamine and catecholamine. A previous study reported that tyrosine was highly associated with the risk of developing diabetes [37]. Elevated tyrosine level was also found to be associated with subclinical atherosclerosis and the development of CAD [38]. Our study demonstrated that tyrosine predicted an increased risk of AP or DVT, and was suggestively associated with a higher risk of CHF or PE in the European population. Moreover, the other non-essential AA, alanine, showed a protective effect on AP in the European population. However, conflicting associations were reported by previous studies in which alanine levels were positively associated with CVD or major cardiovascular events [39–41]. We speculated that this disparity may be ascribed to two reasons. First, it was difficult to infer causality by observational study, which demonstrated that the increased alanine level in previous studies may also be the result of myocardial ischemia episodes rather than a risk factor or that it was merely a biomarker of these disorders. Second, as one study reported, alanine has dual behaviors on CVDs [42]. However, our study only observed its protective role. It is worthwhile to explore the non-linear relationship between alanine and CVDs in future. In brief, the mechanisms underlying the observed effects of tyrosine and alanine remain to be elucidated. In-depth studies are warranted to validate our findings and clarify the detailed mechanisms.

This MR study had several strengths. First, to the best of our knowledge, this is the first MR study that systematically examines the causal effects of 20 genetically encoded AAs on CVDs. Second, we conducted a trans-ancestry MR analysis in both European and EAS populations, and evidences of the causal effects of AAs on different ethnic groups were well provided. It should be noted that our study also had some limitations. First, we did not observe the same causal relationships in European and EAS populations, which was probably due to the limited sample size or genetic variants for the GWAS of AAs in different ethnicities. In addition, the varying genetic background and risk factors across European and EAS populations may also contribute to different findings. Therefore, interpretations should be made with caution in different ethnic groups. Second, due to insufficient IVs, the causal relationships between several AAs and CVDs were not assessed. Future studies need to address this limitation. Third, owing to the absence of datasets, the pleiotropic effects of other factors, such as the metabolites of AAs and the intake of AA-containing foods, were not totally balanced. However, the selection of IVs was performed according to strict criteria to meet the basic assumptions of the MR approach, and no heterogeneity and horizontal pleiotropy among IVs were observed.

## Conclusions

Using the TSMR approach, this study demonstrated significant causal effects of AAs on CVDs in European and EAS populations, respectively. These findings suggested a promising perspective for the prevention and therapeutic targeting of AA metabolism in CVDs. Additional experimental studies and clinical trials are needed to explore the underlying mechanisms and confirm whether the risks can be modified through various intervention methods.

### Supplementary Information

**Additional file 1: Table S1.** Source of GWAS summary statistics. **Table S2.** Instrumental variables for AAs used in this study. **Table S3.** Causal relationships between AAs and CVDs with suggestive significance in European population. **Table S4.** Causal relationships between AAs and CVDs with suggestive significance in EAS population.

## Data Availability

The datasets generated and/or analyzed for this study are available in the MRC IEU OpenGWAS platform (https://gwas.mrcieu.ac.uk/), the Biobank Japan project (https://biosciencedbc.jp and https://pheweb.jp/), the TMM BirThree study and the TMM CommCohort study (https://jmorp.megabank.tohoku.ac.jp/), and a GWAS in Chinese individuals (https://ftp.cngb.org/pub/CNSA/data2/CNP0000794/metabolite/).

## Abbreviations

AA: Amino acid
AP: Angina pectoris
BCAA: Branched chain amino acid
CAD: Coronary artery disease
CHF: Chronic heart failure
CI: Confidence interval
CVD: Cardiovascular disease
DVT: Deep vein thrombosis
EAS: East Asian
GWAS: Genome-wide association study
ICF: Intracerebral hemorrhage
IEU: Integrative Epidemiology Unit
IS: Ischemic stroke
IV: Instrumental variable
IVW-MRE: Inverse variance weighting with multiplicative random effect
LD: Linkage disequilibrium
MI: Myocardial infarction
MR: Mendelian randomization
NMR: Nuclear magnetic resonance
OR: Odds ratio
PAD: Peripheral arterial disease
PE: Pulmonary embolism
SNP: Single-nucleotide polymorphism
TSMR: Two-sample Mendelian randomization
UKB: UK Biobank
UPLC‒MS/MS: Ultra-high performance liquid chromatography-tandem mass spectrometry
VTE: Venous thromboembolism

## References

[1] Townsend N, Kazakiewicz D, Lucy Wright F, Timmis A, Huculeci R, Torbica A, Gale CP, Achenbach S, Weidinger F, Vardas P (2022). Epidemiology of cardiovascular disease in Europe. Nat Rev Cardiol.

[2] Roth GA, Mensah GA, Johnson CO, Addolorato G, Ammirati E, Baddour LM, Barengo NC, Beaton AZ, Benjamin EJ, Benziger CP (2020). Global burden of cardiovascular diseases and risk factors, 1990–2019: update from the GBD 2019 study. J Am Coll Cardiol.

[3] Teo KK, Rafiq T (2021). Cardiovascular risk factors and prevention: a perspective from developing countries. Can J Cardiol.

[4] Nitz K, Lacy M, Atzler D (2019). Amino acids and their metabolism in atherosclerosis. Arterioscler Thromb Vasc Biol.

[5] Durante W (2020). Amino acids in circulatory function and health. Adv Exp Med Biol.

[6] Grosse GM, Schwedhelm E, Worthmann H, Choe CU (2020). Arginine derivatives in cerebrovascular diseases: mechanisms and clinical implications. Int J Mol Sci.

[7] Oberkersch RE, Santoro MM (2019). Role of amino acid metabolism in angiogenesis. Vascul Pharmacol.

[8] Yu E, Ruiz-Canela M, Hu FB, Clish CB, Corella D, Salas-Salvado J, Hruby A, Fito M, Liang L, Toledo E (2017). Plasma arginine/asymmetric dimethylarginine ratio and incidence of cardiovascular events: a case-cohort study. J Clin Endocrinol Metab.

[9] Yu E, Ruiz-Canela M, Guasch-Ferre M, Zheng Y, Toledo E, Clish CB, Salas-Salvado J, Liang L, Wang DD, Corella D (2017). Increases in plasma tryptophan are inversely associated with incident cardiovascular disease in the prevencion con dieta mediterranea (PREDIMED) study. J Nutr.

[10] Ruiz-Canela M, Toledo E, Clish CB, Hruby A, Liang L, Salas-Salvado J, Razquin C, Corella D, Estruch R, Ros E (2016). Plasma branched-chain amino acids and incident cardiovascular disease in the PREDIMED trial. Clin Chem.

[11] Magnusson M, Lewis GD, Ericson U, Orho-Melander M, Hedblad B, Engstrom G, Ostling G, Clish C, Wang TJ, Gerszten RE, Melander O (2013). A diabetes-predictive amino acid score and future cardiovascular disease. Eur Heart J.

[12] Wang Y, Huang K, Liu F, Lu X, Huang J, Gu D (2022). Association of circulating branched-chain amino acids with risk of cardiovascular disease: a systematic review and meta-analysis. Atherosclerosis.

[13] Benn M, Nordestgaard BG, Frikke-Schmidt R, Tybjaerg-Hansen A (2017). Low LDL cholesterol, PCSK9 and HMGCR genetic variation, and risk of Alzheimer’s disease and Parkinson’s disease: mendelian randomisation study. BMJ.

[14] Larsson SC, Mason AM, Back M, Klarin D, Damrauer SM, Million Veteran P, Michaelsson K, Burgess S (2020). Genetic predisposition to smoking in relation to 14 cardiovascular diseases. Eur Heart J.

[15] Elsworth B, Lyon M, Alexander T, Liu Y, Matthews P, Hallett J, Bates P, Palmer T, Haberland V, Smith GD (2020). The MRC IEU OpenGWAS data infrastructure. BioRxiv.

[16] Kettunen J, Demirkan A, Wurtz P, Draisma HH, Haller T, Rawal R, Vaarhorst A, Kangas AJ, Lyytikainen LP, Pirinen M (2016). Genome-wide study for circulating metabolites identifies 62 loci and reveals novel systemic effects of LPA. Nat Commun.

[17] Shin SY, Fauman EB, Petersen AK, Krumsiek J, Santos R, Huang J, Arnold M, Erte I, Forgetta V, Yang TP (2014). An atlas of genetic influences on human blood metabolites. Nat Genet.

[18] Sakaue S, Kanai M, Tanigawa Y, Karjalainen J, Kurki M, Koshiba S, Narita A, Konuma T, Yamamoto K, Akiyama M (2021). A cross-population atlas of genetic associations for 220 human phenotypes. Nat Genet.

[19] Liu X, Tong X, Zou Y, Lin X, Zhao H, Tian L, Jie Z, Wang Q, Zhang Z, Lu H (2022). Mendelian randomization analyses support causal relationships between blood metabolites and the gut microbiome. Nat Genet.

[20] van der Harst P, Verweij N (2018). Identification of 64 novel genetic loci provides an expanded view on the genetic architecture of coronary artery disease. Circ Res.

[21] Shah S, Henry A, Roselli C, Lin H, Sveinbjornsson G, Fatemifar G, Hedman AK, Wilk JB, Morley MP, Chaffin MD (2020). Genome-wide association and mendelian randomisation analysis provide insights into the pathogenesis of heart failure. Nat Commun.

[22] Malik R, Chauhan G, Traylor M, Sargurupremraj M, Okada Y, Mishra A, Rutten-Jacobs L, Giese AK, van der Laan SW, Gretarsdottir S (2018). Multiancestry genome-wide association study of 520,000 subjects identifies 32 loci associated with stroke and stroke subtypes. Nat Genet.

[23] Koyama S, Ito K, Terao C, Akiyama M, Horikoshi M, Momozawa Y, Matsunaga H, Ieki H, Ozaki K, Onouchi Y (2020). Population-specific and trans-ancestry genome-wide analyses identify distinct and shared genetic risk loci for coronary artery disease. Nat Genet.

[24] Bowden J, Spiller W, Del Greco MF, Sheehan N, Thompson J, Minelli C, Davey Smith G (2018). Improving the visualization, interpretation and analysis of two-sample summary data Mendelian randomization via the radial plot and radial regression. Int J Epidemiol..

[25] Tobias DK, Lawler PR, Harada PH, Demler OV, Ridker PM, Manson JE, Cheng S, Mora S (2018). Circulating branched-chain amino acids and incident cardiovascular disease in a prospective cohort of US women. Circ Genom Precis Med.

[26] Lim LL, Lau ESH, Fung E, Lee HM, Ma RCW, Tam CHT, Wong WKK, Ng ACW, Chow E, Luk AOY (2020). Circulating branched-chain amino acids and incident heart failure in type 2 diabetes: the Hong Kong diabetes register. Diabetes Metab Res Rev.

[27] Zhenyukh O, Civantos E, Ruiz-Ortega M, Sanchez MS, Vazquez C, Peiro C, Egido J, Mas S (2017). High concentration of branched-chain amino acids promotes oxidative stress, inflammation and migration of human peripheral blood mononuclear cells via mTORC1 activation. Free Radic Biol Med.

[28] Zhenyukh O, Gonzalez-Amor M, Rodrigues-Diez RR, Esteban V, Ruiz-Ortega M, Salaices M, Mas S, Briones AM, Egido J (2018). Branched-chain amino acids promote endothelial dysfunction through increased reactive oxygen species generation and inflammation. J Cell Mol Med.

[29] Xu Y, Jiang H, Li L, Chen F, Liu Y, Zhou M, Wang J, Jiang J, Li X, Fan X (2020). Branched-chain amino acid catabolism promotes thrombosis risk by enhancing tropomodulin-3 propionylation in platelets. Circulation.

[30] Mishra RC, Tripathy S, Desai KM, Quest D, Lu Y, Akhtar J, Gopalakrishnan V (2008). Nitric oxide synthase inhibition promotes endothelium-dependent vasodilatation and the antihypertensive effect of l-serine. Hypertension.

[31] Diaz-Flores M, Cruz M, Duran-Reyes G, Munguia-Miranda C, Loza-Rodriguez H, Pulido-Casas E, Torres-Ramirez N, Gaja-Rodriguez O, Kumate J, Baiza-Gutman LA, Hernandez-Saavedra D (2013). Oral supplementation with glycine reduces oxidative stress in patients with metabolic syndrome, improving their systolic blood pressure. Can J Physiol Pharmacol.

[32] Dietrich S, Floegel A, Weikert C, Prehn C, Adamski J, Pischon T, Boeing H, Drogan D (2016). Identification of serum metabolites associated with incident hypertension in the European prospective investigation into cancer and nutrition-potsdam study. Hypertension.

[33] Ding Y, Svingen GF, Pedersen ER, Gregory JF, Ueland PM, Tell GS, Nygard OK (2015). Plasma glycine and risk of acute myocardial infarction in patients with suspected stable angina pectoris. J Am Heart Assoc.

[34] Leipnitz G, da Silva Lde B, Fernandes CG, Seminotti B, Amaral AU, Dutra-Filho CS, Wajner M (2010). d-Serine administration provokes lipid oxidation and decreases the antioxidant defenses in rat striatum. Int J Dev Neurosci.

[35] Maralani MN, Movahedian A, Javanmard Sh H (2012). Antioxidant and cytoprotective effects of l-serine on human endothelial cells. Res Pharm Sci.

[36] Wang W, Wu Z, Dai Z, Yang Y, Wang J, Wu G (2013). Glycine metabolism in animals and humans: implications for nutrition and health. Amino Acids.

[37] Wang TJ, Larson MG, Vasan RS, Cheng S, Rhee EP, McCabe E, Lewis GD, Fox CS, Jacques PF, Fernandez C (2011). Metabolite profiles and the risk of developing diabetes. Nat Med.

[38] Wurtz P, Raiko JR, Magnussen CG, Soininen P, Kangas AJ, Tynkkynen T, Thomson R, Laatikainen R, Savolainen MJ, Laurikka J (2012). High-throughput quantification of circulating metabolites improves prediction of subclinical atherosclerosis. Eur Heart J.

[39] Rizza S, Copetti M, Rossi C, Cianfarani MA, Zucchelli M, Luzi A, Pecchioli C, Porzio O, Di Cola G, Urbani A (2014). Metabolomics signature improves the prediction of cardiovascular events in elderly subjects. Atherosclerosis.

[40] Ameta K, Gupta A, Ameta D, Sethi R, Kumar D, Ahmad I, Mahdi AA (2016). 1H NMR-derived metabolomics of filtered serum of myocardial ischemia in unstable angina patients. Clin Chim Acta.

[41] Deidda M, Noto A, Cadeddu Dessalvi C, Andreini D, Andreotti F, Ferrannini E, Latini R, Maggioni AP, Magnoni M, Maseri A (2021). Metabolomic correlates of coronary atherosclerosis, cardiovascular risk, both or neither. Results of the 2 × 2 phenotypic CAPIRE study. Int J Cardiol.

[42] Deidda M, Piras C, Cadeddu Dessalvi C, Congia D, Locci E, Ascedu F, De Candia G, Cadeddu M, Lai G, Pirisi R (2017). Blood metabolomic fingerprint is distinct in healthy coronary and in stenosing or microvascular ischemic heart disease. J Transl Med.

