# Supplementary material for: Causal relationships of circulating amino acids with cardiovascular disease: a trans-ancestry Mendelian randomization analysis
Source: J Transl Med. 2023 Oct 7;21:699. doi: 10.1186/s12967-023-04580-y (PMC10559604; doi:10.1186/s12967-023-04580-y)
Supplement: Supplementary file 1 — Additional file 1: Table S1. Source of GWAS summary statistics. Table S2. Instrumental variables for AAs used in this study. Table S3. Causal relationships between AAs and CVDs with suggestive significance in European population. Table S4. Causal relationships between AAs and CVDs with suggestive significance in EAS population. [file 12967_2023_4580_MOESM1_ESM.docx]

**Additional file 1**

**Table S1. Source of GWAS summary statistics**

| **Trait** | **European** | | | **East Asian** | | |
| --- | --- | --- | --- | --- | --- | --- |
|  | **Sample size**  **(case/control)** | **No. of SNPs** | **Reference** | **Sample size**  **(case/control)** | **No. of SNPs** | **Reference** |
| **AA** |  |  |  |  |  |  |
| Alanine | 24,796 | 12,091,566 | Kettunen J, et al. 2016^1^ | 7,842 | 8,084,830 | Sakaue S, et al. 2021^2^ |
| Arginine | 7,528 | 2,545,579 | Shin, S et al. 2014^3^ | 7,830 | 8,084,524 | Sakaue S, et al. 2021^2^ |
| Asparagine | 7,761 | 2,545,507 | Shin, S et al. 2014^3^ | 7,828 | 8,084,547 | Sakaue S, et al. 2021^2^ |
| Aspartic acid | 7,721 | 2,545,425 | Shin, S et al. 2014^3^ | 1,680 | 9,913,984 | Liu X, et al. 2022^4^ |
| Cysteine | 7,692 | 2,545,727 | Shin, S et al. 2014^3^ | 7,838 | 8,084,535 | Sakaue S, et al. 2021^2^ |
| Glutamic acid | 7,804 | 2,545,537 | Shin, S et al. 2014^3^ | 7,841 | 8,084,588 | Sakaue S, et al. 2021^2^ |
| Glutamine | 24,462 | 11,976,419 | Kettunen J, et al. 2016^1^ | 7,830 | 8,084,576 | Sakaue S, et al. 2021^2^ |
| Glycine | 7,802 | 2,545,539 | Shin, S et al. 2014^3^ | 7,843 | 8,084,826 | Sakaue S, et al. 2021^2^ |
| Histidine | 19,244 | 11,811,025 | Kettunen J, et al. 2016^1^ | 7,814 | 8,084,438 | Sakaue S, et al. 2021^2^ |
| Isoleucine | 24,776 | 12,076,452 | Kettunen J, et al. 2016^1^ | 7,839 | 8,084,794 | Sakaue S, et al. 2021^2^ |
| Leucine | 24,728 | 12,078,191 | Kettunen J, et al. 2016^1^ | 7,838 | 8,084,775 | Sakaue S, et al. 2021^2^ |
| Lysine | 7,812 | 2,545,686 | Shin, S et al. 2014^3^ | 7,841 | 8,084,811 | Sakaue S, et al. 2021^2^ |
| Methionine | 7,795 | 2,545,691 | Shin, S et al. 2014^3^ | 7,822 | 8,084,735 | Sakaue S, et al. 2021^2^ |
| Phenylalanine | 22,663 | 12,042,964 | Kettunen J, et al. 2016^1^ | 7,832 | 8,084,693 | Sakaue S, et al. 2021^2^ |
| Proline | 7,816 | 2,545,669 | Shin, S et al. 2014^3^ | 7,833 | 8,084,770 | Sakaue S, et al. 2021^2^ |
| Serine | 7,796 | 2,545,555 | Shin, S et al. 2014^3^ | 7,828 | 8,084,735 | Sakaue S, et al. 2021^2^ |
| Threonine | 6,020 | 2,545,896 | Shin, S et al. 2014^3^ | 7,831 | 8,084,343 | Sakaue S, et al. 2021^2^ |
| Tryptophan | 7,804 | 2,545,641 | Shin, S et al. 2014^3^ | 7,829 | 8,084,446 | Sakaue S, et al. 2021^2^ |
| Tyrosine | 24,925 | 12,090,785 | Kettunen J, et al. 2016^1^ | 7,839 | 8,084,654 | Sakaue S, et al. 2021^2^ |
| Valine | 24,900 | 12,092,490 | Kettunen J, et al. 2016^1^ | 7,842 | 8,084,799 | Sakaue S, et al. 2021^2^ |
| **CVD** |  |  |  |  |  |  |
| Coronary artery disease | 547,261 (122,733/424,528) | 7,934,254 | van der Harst P, et al 2018^5^ | 168,228 (25,892/142,336) | 13,748,712 | Koyama S, et al. 2020^6^ |
| Myocardial infarction | 337,199 (3,927/333,272) | 10,894,596 | Elsworth B, et al. 2020^7^ | 161,206 (14,992/146,214) | 13,433,236 | Sakaue S, et al. 2021^2^ |
| Angina pectoris | 337,199 (4,837/332,362) | 10,894,596 | Elsworth B, et al. 2020^7^ | 159,165 (14,007/145,158) | 13,432,806 | Sakaue S, et al. 2021^2^ |
| Chronic heart failure | 977,323 (47,309/930,014) | 7,773,021 | Shah, S, et al. 2020^8^ | 178,726 (10,540/168,186) | 13,436,083 | Sakaue S, et al. 2021^2^ |
| Ischemic stroke | 440,328 (34,217/406,111) | 7,537,579 | Malik, R, et al. 2018^9^ | 174,686 (22,664/152,022) | 13,435,541 | Sakaue S, et al. 2021^2^ |
| Intracerebral hemorrhage | 202,833 (1,687/201,146) | 16,380,393 | Elsworth B, et al. 2020^7^ | 153,478 (1,456/152,022) | 13,431,918 | Sakaue S, et al. 2021^2^ |
| Peripheral arterial disease | 361,194 (1,230/359,964) | 9,637,467 | Elsworth B, et al. 2020^7^ | 177,713 (4,112/173,601) | 13,435,927 | Sakaue S, et al. 2021^2^ |
| Venous thromboembolism | 361,194 (4,620/356,574) | 11,901,177 | Elsworth B, et al. 2020^7^ | NA | NA | NA |
| Deep vein thrombosis | 337,159 (6,767/330,392) | 10,894,596 | Elsworth B, et al. 2020^7^ | NA | NA | NA |
| Pulmonary embolism | 337,159 (2,801/334,358) | 10,894,596 | Elsworth B, et al. 2020^7^ | NA | NA | NA |

AA indicates amino acid; CVD, cardiovascular disease; GWAS, genome-wide association study; NA, no available; SNP, single nucleotide polymorphism.

**Table S2. Instrumental variables for AAs used in this study**

| **Exposure** | **SNP** | **Position** | **Gene** | **EA** | **OA** | **EAF** | **β** | **SE** | ***P*-value** | **F statistics** |
| --- | --- | --- | --- | --- | --- | --- | --- | --- | --- | --- |
| **European** |  |  |  |  |  |  |  |  |  |  |
| Alanine | rs1260326 | 2:27730940 | GCKR | C | T | 0.6388 | -0.1046 | 0.0099 | 7.40E-26 | 110.7 |
|  | rs2160387 | 2:65220910 | SLC1A4 | C | T | 0.4032 | -0.071 | 0.0096 | 1.49E-13 | 54.67 |
|  | rs12578760 | 12:47105117 | NA | T | C | 0.1448 | 0.0797 | 0.0131 | 1.36E-09 | 36.79 |
|  | rs4554975 | 12:47201814 | SLC38A4 | G | A | 0.6441 | -0.0691 | 0.0096 | 6.12E-13 | 51.88 |
|  | rs2694917 | 12:56912864 | RBMS2 | C | T | 0.1685 | 0.086 | 0.013 | 3.68E-11 | 43.84 |
|  | rs149191093 | 16:70341358 | DDX19B | T | C | 0.0432 | -0.1619 | 0.0245 | 4.05E-11 | 43.66 |
| Glutamine | rs1260326 | 2:27730940 | GCKR | C | T | 0.6390 | 0.0617 | 0.0101 | 9.87E-10 | 37.41 |
|  | rs6729711 | 2:191720765 | NA | A | G | 0.1813 | -0.0696 | 0.0124 | 2.23E-08 | 31.33 |
|  | rs7078003 | 10:99359412 | HOGA1 | T | C | 0.1959 | 0.0739 | 0.0117 | 2.96E-10 | 39.76 |
|  | rs7952320 | 11:8250143 | LMO1 | C | G | 0.5338 | 0.0538 | 0.0094 | 9.78E-09 | 32.93 |
|  | rs12306007 | 12:47184042 | SLC38A4 | T | C | 0.1401 | 0.0794 | 0.0134 | 3.63E-09 | 34.87 |
|  | rs2657879 | 12:56865338 | GLS2, SPRYD4 | G | A | 0.1765 | -0.2214 | 0.0125 | 3.30E-70 | 314.03 |
| Histidine | rs3733402 | 4:187158034 | KLKB1 | A | G | 0.5680 | 0.0813 | 0.0104 | 9.14E-15 | 60.84 |
|  | rs1801020 | 5:176836532 | F12 | G | A | 0.7279 | 0.0777 | 0.0118 | 5.83E-11 | 43.43 |
|  | rs7954638 | 12:96314795 | CCDC38 | A | C | 0.4824 | -0.082 | 0.0105 | 7.25E-15 | 61.3 |
|  | rs73216231 | 12:96384437 | HAL | T | A | 0.1176 | 0.1208 | 0.0161 | 8.94E-14 | 56.3 |
|  | rs1998848 | 14:21492229 | NDRG2, MIR6717 | A | G | 0.0540 | 0.1475 | 0.0236 | 4.91E-10 | 39.21 |
| Leucine | rs1260326 | 2:27730940 | GCKR | C | T | 0.6390 | -0.0798 | 0.0099 | 1.07E-15 | 64.5 |
|  | rs17789027 | 4:89181841 | PPM1K | G | A | 0.3843 | 0.1086 | 0.0095 | 6.16E-30 | 129.55 |
|  | rs12325419 | 16:70368909 | DDX19B, LOC100506083 | A | G | 0.1202 | -0.0821 | 0.015 | 4.55E-08 | 29.99 |
| Phenylalanine | rs182695896 | 4:74813227 | NA | C | A | 0.0181 | 0.2453 | 0.0401 | 1.09E-09 | 37.4 |
|  | rs4253238 | 4:187148387 | KLKB1 | T | C | 0.5551 | 0.0673 | 0.0101 | 2.53E-11 | 44.81 |
|  | rs2731672 | 5:176842474 | NA | C | T | 0.7400 | 0.0949 | 0.0116 | 3.85E-16 | 66.75 |
|  | rs1718309 | 12:103242396 | PAH | G | A | 0.6026 | -0.0772 | 0.0097 | 2.50E-15 | 63.04 |
| Proline | rs11802885 | 1:113896872 | NA | A | T | 0.3634 | 0.0098 | 0.0018 | 2.33E-08 | 29.64 |
|  | rs5747934 | 22:18915282 | PRODH | T | C | 0.0468 | 0.0715 | 0.0051 | 2.00E-44 | 196.55 |
|  | rs2518802 | 22:18975345 | DGCR5 | C | A | 0.0970 | 0.0534 | 0.0033 | 4.15E-59 | 261.85 |
| Serine | rs1163251 | 1:120209755 | NA | C | T | 0.4000 | -0.0188 | 0.0017 | 7.05E-27 | 122.3 |
|  | rs715 | 2:211543055 | CPS1 | C | T | 0.2889 | 0.0223 | 0.0024 | 2.69E-21 | 86.34 |
|  | rs4947534 | 7:56079094 | PSPH | C | T | 0.7521 | 0.0183 | 0.0024 | 1.96E-14 | 58.14 |
| Tryptophan | rs284191 | 1:92236048 | TGFBR3 | G | A | 0.3850 | 0.006 | 0.001 | 1.97E-09 | 36 |
|  | rs7584842 | 2:11756260 | GREB1 | C | T | 0.4623 | 0.005 | 0.0009 | 4.15E-08 | 30.86 |
|  | rs4306882 | 3:21062584 | NA | T | G | 0.6153 | -0.0057 | 0.0009 | 2.52E-10 | 40.11 |
|  | rs710580 | 3:189809163 | P3H2 | A | C | 0.3566 | -0.005 | 0.0009 | 3.57E-08 | 30.86 |
|  | rs972459 | 3:191855497 | FGF12 | C | T | 0.4213 | -0.005 | 0.0009 | 1.97E-08 | 30.86 |
|  | rs4695138 | 4:45956180 | NA | T | A | 0.4648 | -0.0052 | 0.0009 | 8.00E-09 | 33.38 |
|  | rs13122250 | 4:156808288 | NA | T | C | 0.5542 | 0.0062 | 0.0009 | 8.95E-12 | 47.46 |
|  | rs4615256 | 5:62700867 | NA | G | A | 0.4673 | -0.0049 | 0.0009 | 4.99E-08 | 29.64 |
|  | rs1373962 | 5:83527862 | EDIL3 | C | T | 0.4027 | -0.005 | 0.0009 | 2.71E-08 | 30.86 |
|  | rs1016522 | 5:143193326 | HMHB1 | A | G | 0.5803 | 0.0058 | 0.0009 | 1.59E-10 | 41.53 |
|  | rs4958379 | 5:154010041 | NA | A | G | 0.4330 | -0.005 | 0.0009 | 2.18E-08 | 30.86 |
|  | rs1559063 | 5:172348594 | ERGIC1 | G | C | 0.3791 | -0.0052 | 0.0009 | 7.82E-09 | 33.38 |
|  | rs6901004 | 6:111554822 | SLC16A10 | G | C | 0.4258 | 0.0061 | 0.0009 | 1.08E-11 | 45.94 |
|  | rs6935961 | 6:125636350 | NA | G | A | 0.4422 | 0.0056 | 0.0009 | 3.75E-10 | 38.72 |
|  | rs38271 | 7:14307031 | DGKB | G | A | 0.4075 | 0.0051 | 0.0009 | 1.19E-08 | 32.11 |
|  | rs7463805 | 8:110753672 | NA | T | C | 0.5213 | -0.0053 | 0.0009 | 4.60E-09 | 34.68 |
|  | rs6480970 | 10:54568664 | NA | A | G | 0.6159 | -0.0049 | 0.0009 | 4.29E-08 | 29.64 |
|  | rs603446 | 11:116654435 | ZPR1 | T | C | 0.4448 | 0.0051 | 0.0009 | 1.38E-08 | 32.11 |
|  | rs9511152 | 13:19596767 | NA | A | G | 0.5590 | -0.005 | 0.0009 | 2.98E-08 | 30.86 |
|  | rs2111118 | 16:54049153 | FTO | C | T | 0.3793 | -0.0051 | 0.0009 | 1.21E-08 | 32.11 |
| Tyrosine | rs14399 | 6:111543944 | SLC16A10 | A | C | 0.3899 | -0.0967 | 0.0094 | 1.43E-24 | 104.82 |
|  | rs1169289 | 12:121416622 | HNF1A | G | C | 0.4686 | 0.0526 | 0.0094 | 1.95E-08 | 31.59 |
|  | rs150851429 | 16:71625831 | NA | C | G | 0.0163 | 0.4108 | 0.045 | 6.68E-20 | 83.52 |
|  | rs9931717 | 16:71813959 | AP1G1 | T | C | 0.3534 | -0.0631 | 0.0096 | 5.64E-11 | 43.01 |
|  | rs28663143 | 17:20895571 | NA | C | T | 0.2944 | -0.06 | 0.0102 | 4.81E-09 | 34.31 |
| Valine | rs10211524 | 2:65208074 | NA | A | G | 0.4099 | 0.0864 | 0.0094 | 5.24E-20 | 84.01 |
|  | rs7655059 | 4:89149144 | ABCG2 | G | C | 0.2177 | -0.0685 | 0.0112 | 8.91E-10 | 37.61 |
|  | rs9637599 | 4:89206230 | PPM1K, LOC105369192 | C | A | 0.4696 | 0.1139 | 0.0092 | 1.67E-35 | 154.83 |
|  | rs2072560 | 11:116661826 | APOA5, ZPR1 | C | T | 0.9285 | 0.1047 | 0.0177 | 3.28E-09 | 35.06 |
|  | rs7406661 | 17:7063667 | NA | C | T | 0.2434 | 0.0791 | 0.0127 | 5.35E-10 | 38.6 |
| **East Asian** |  |  |  |  |  |  |  |  |  |  |
| Glycine | rs3755181 | 2:211342324 | CPS1, LANCL1 | C | T | 0.0260 | 0.2901 | 0.0514 | 1.68E-08 | 31.9 |
|  | rs76079872 | 2:211441311 | CPS1 | T | C | 0.0212 | 0.4049 | 0.0554 | 2.87E-13 | 53.48 |
|  | rs7599931 | 2:211517423 | CPS1 | G | T | 0.2161 | 0.1965 | 0.0192 | 2.52E-24 | 104.26 |
|  | rs1047891 | 2:211540507 | CPS1 | A | C | 0.1548 | 0.9149 | 0.0192 | 1.00E-200 | 2260.11 |
|  | rs11683146 | 2:211640124 | NA | G | A | 0.0647 | 0.3837 | 0.0321 | 1.38E-32 | 142.6 |
|  | rs60978506 | 2:211836582 | NA | T | C | 0.0214 | 0.6125 | 0.0551 | 1.59E-28 | 123.72 |
|  | rs4673578 | 2:211874258 | NA | G | A | 0.3167 | 0.1254 | 0.0171 | 2.44E-13 | 53.8 |
|  | rs183886547 | 2:212138885 | NA | T | A | 0.0292 | 0.4214 | 0.0485 | 4.55E-18 | 75.43 |
|  | rs10934753 | 3:125906179 | ALDH1L1-AS2 | A | G | 0.2744 | 0.1502 | 0.0176 | 1.48E-17 | 73.09 |
|  | rs10251184 | 7:56083359 | PSPH | T | A | 0.5968 | 0.1377 | 0.0163 | 3.01E-17 | 71.67 |
|  | rs79063043 | 16:81131790 | PKD1L2, GCSH | C | G | 0.1829 | -0.1157 | 0.0207 | 2.48E-08 | 31.14 |
|  | rs118049419 | 16:81150935 | PKD1L2 | A | C | 0.0251 | -0.4917 | 0.0512 | 1.02E-21 | 92.22 |
| Histidine | rs1047891 | 2:211540507 | CPS1 | A | C | 0.1548 | -0.1885 | 0.0218 | 5.87E-18 | 74.93 |
|  | rs143734418 | 12:96070262 | NTN4, PGAM1P5 | A | G | 0.0346 | 0.2976 | 0.0449 | 3.58E-11 | 43.96 |
|  | rs12371207 | 12:96162661 | NTN4 | A | G | 0.6168 | 0.0922 | 0.0165 | 2.27E-08 | 31.31 |
|  | rs78676864 | 12:96392507 | LTA4H, HAL | G | A | 0.0799 | 0.27 | 0.0294 | 4.73E-20 | 84.55 |
|  | rs2247570 | 12:96422377 | LTA4H | C | T | 0.1195 | -0.1705 | 0.0246 | 4.57E-12 | 48.01 |
|  | rs74036552 | 14:21486590 | NDRG2, MIR6717 | T | C | 0.1909 | 0.2282 | 0.0201 | 9.33E-30 | 129.44 |
|  | rs10138807 | 14:21492310 | NDRG2, MIR6717 | T | C | 0.5190 | 0.1321 | 0.0159 | 9.21E-17 | 69.44 |
| Phenylalanine | rs7666826 | 4:54720109 | NA | T | A | 0.1476 | -0.1258 | 0.0225 | 2.41E-08 | 31.2 |
|  | rs148186416 | 12:102376053 | NA | G | T | 0.0219 | 0.3615 | 0.0564 | 1.52E-10 | 41.11 |
|  | rs118034789 | 12:102475119 | NUP37 | C | T | 0.0727 | 0.1739 | 0.0307 | 1.53E-08 | 32.09 |
|  | rs138662004 | 12:103004989 | NA | G | A | 0.0514 | 0.2605 | 0.036 | 4.71E-13 | 52.5 |
|  | rs10860912 | 12:103164355 | NA | C | T | 0.3542 | -0.1296 | 0.0168 | 1.47E-14 | 59.36 |
|  | rs870072 | 12:103243177 | PAH | T | C | 0.0852 | -0.2687 | 0.0283 | 2.76E-21 | 90.23 |
|  | rs118092776 | 12:103306579 | PAH | T | C | 0.0531 | 1.0049 | 0.0337 | 1.14E-185 | 891.8 |
|  | rs7312875 | 12:103410438 | NA | G | A | 0.6781 | 0.0991 | 0.0172 | 7.87E-09 | 33.38 |
|  | rs10778260 | 12:103925240 | NA | C | T | 0.2561 | 0.1011 | 0.0182 | 2.93E-08 | 30.81 |
| Proline | rs3761097 | 19:36290977 | PRODH2, LINC01529 | T | C | 0.1693 | 0.137 | 0.0214 | 1.73E-10 | 40.86 |
|  | rs5747933 | 22:18910355 | PRODH | T | G | 0.1474 | 0.6664 | 0.0212 | 1.00E-200 | 989.58 |
|  | rs11912152 | 22:18912779 | PRODH | C | T | 0.0204 | -0.5037 | 0.0557 | 1.91E-19 | 81.76 |
|  | rs76411026 | 22:19020460 | DGCR2, DGCR5 | T | G | 0.0207 | 0.5423 | 0.0578 | 8.00E-21 | 88.1 |
|  | rs9618617 | 22:19584197 | NA | G | A | 0.2268 | -0.105 | 0.019 | 3.69E-08 | 30.37 |
| Serine | rs2950835 | 2:27750545 | GCKR | G | A | 0.4291 | 0.104 | 0.0161 | 1.16E-10 | 41.65 |
|  | rs1047891 | 2:211540507 | CPS1 | A | C | 0.1550 | 0.3061 | 0.0216 | 3.94E-45 | 201.29 |
|  | rs10251184 | 7:56083359 | PSPH | T | A | 0.5967 | 0.2245 | 0.0162 | 2.15E-43 | 193.14 |
|  | rs1057603 | 7:56108410 | PSPH | A | T | 0.2316 | 0.1467 | 0.0188 | 6.56E-15 | 60.97 |
|  | rs11771965 | 7:57545360 | NA | C | G | 0.6331 | 0.0918 | 0.0164 | 2.47E-08 | 31.15 |

AA indicates amino acid; EA, effect allele; EAF, effect allele frequency; NA, no available; OA, other allele; SE, standard error; SNP, single nucleotide polymorphism.

**Table S3. Causal relationships between AAs and CVDs with suggestive significance in European population**

| **Outcome** | **Exposure** | **Method** | **OR (95% CI)** | ***P*** | **No. of SNPs** | ***P_heterogeneity_*** | ***P_pleiotropy_*** |
| --- | --- | --- | --- | --- | --- | --- | --- |
| Coronary artery disease | Histidine | IVW-MRE | 0.941 (0.898, 0.986) | 1.05E-02 | 4 | 0.8819 | 0.7797 |
|  |  | MR Egger | 0.800 (0.296, 2.167) | 7.04E-01 |  |  |  |
|  |  | Weighted mode | 0.931 (0.813, 1.066) | 3.76E-01 |  |  |  |
|  |  | Weighted median | 0.933 (0.828, 1.051) | 2.52E-01 |  |  |  |
| Angina pectoris | Histidine | IVW-MRE | 0.995 (0.991, 0.998) | 4.25E-03 | 5 | 0.422 | 0.8416 |
|  |  | MR Egger | 0.997 (0.975, 1.021) | 8.37E-01 |  |  |  |
|  |  | Weighted mode | 0.993 (0.987, 0.999) | 8.44E-02 |  |  |  |
|  |  | Weighted median | 0.994 (0.989, 0.998) | 7.95E-03 |  |  |  |
|  | Leucine | IVW-MRE | 1.004 (1.001, 1.008) | 2.58E-02 | 3 | 0.4307 | 0.8993 |
|  |  | MR Egger | 1.001 (0.968, 1.036) | 9.49E-01 |  |  |  |
|  |  | Weighted mode | 1.005 (0.999, 1.010) | 2.20E-01 |  |  |  |
|  |  | Weighted median | 1.005 (1.000, 1.009) | 4.90E-02 |  |  |  |
| Peripheral arterial disease | Leucine | IVW-MRE | 1.002 (1.002, 1.003) | 6.07E-12 | 3 | 0.9018 | 0.729 |
|  |  | MR Egger | 0.999 (0.987, 1.012) | 9.24E-01 |  |  |  |
|  |  | Weighted mode | 1.002 (0.999, 1.004) | 3.11E-01 |  |  |  |
|  |  | Weighted median | 1.002 (1.000, 1.004) | 4.19E-02 |  |  |  |
| Deep vein thrombosis | Leucine | IVW-MRE | 0.996 (0.994, 0.999) | 5.04E-03 | 3 | 0.7427 | 0.6436 |
|  |  | MR Egger | 1.006 (0.975, 1.039) | 7.62E-01 |  |  |  |
|  |  | Weighted mode | 0.998 (0.991, 1.004) | 5.15E-01 |  |  |  |
|  |  | Weighted median | 0.997 (0.992, 1.002) | 3.19E-01 |  |  |  |
| Coronary artery disease | Phenylalanine | IVW-MRE | 0.947 (0.904, 0.992) | 2.07E-02 | 3 | 0.8217 | 0.8217 |
|  |  | MR Egger | 1.055 (0.501, 2.221) | 9.11E-01 |  |  |  |
|  |  | Weighted mode | 0.958 (0.834, 1.100) | 6.03E-01 |  |  |  |
|  |  | Weighted median | 0.952 (0.844, 1.074) | 4.25E-01 |  |  |  |
| Myocardial infarction | Phenylalanine | IVW-MRE | 0.997 (0.996, 0.998) | 3.57E-08 | 4 | 0.9755 | 0.751 |
|  |  | MR Egger | 1.000 (0.986, 1.014) | 9.74E-01 |  |  |  |
|  |  | Weighted mode | 0.997 (0.992, 1.003) | 4.10E-01 |  |  |  |
|  |  | Weighted median | 0.997 (0.993, 1.002) | 2.12E-01 |  |  |  |
| Deep vein thrombosis | Serine | IVW-MRE | 1.028 (1.013, 1.044) | 3.09E-04 | 3 | 0.6019 | 0.7542 |
|  |  | MR Egger | 0.979 (0.772, 1.242) | 8.89E-01 |  |  |  |
|  |  | Weighted mode | 1.022 (0.992, 1.053) | 2.92E-01 |  |  |  |
|  |  | Weighted median | 1.024 (0.998, 1.050) | 7.09E-02 |  |  |  |
| Venous thromboembolism | Serine | IVW-MRE | 1.007 (1.003, 1.012) | 1.29E-03 | 3 | 0.9315 | 0.8774 |
|  |  | MR Egger | 1.026 (0.853, 1.233) | 8.32E-01 |  |  |  |
|  |  | Weighted mode | 1.009 (0.988, 1.032) | 4.86E-01 |  |  |  |
|  |  | Weighted median | 1.009 (0.991, 1.028) | 3.08E-01 |  |  |  |
| Angina pectoris | Tryptophan | IVW-MRE | 0.967 (0.942, 0.992) | 1.03E-02 | 18 | 0.4569 | 0.6221 |
|  |  | MR Egger | 0.883 (0.619, 1.259) | 5.02E-01 |  |  |  |
|  |  | Weighted mode | 0.964 (0.902, 1.031) | 2.98E-01 |  |  |  |
|  |  | Weighted median | 0.967 (0.934, 1.003) | 6.98E-02 |  |  |  |
| Deep vein thrombosis | Tryptophan | IVW-MRE | 1.034 (1.000, 1.069) | 4.92E-02 | 17 | 0.304 | 0.1106 |
|  |  | MR Egger | 1.488 (0.976, 2.268) | 8.46E-02 |  |  |  |
|  |  | Weighted mode | 1.039 (0.962, 1.123) | 3.47E-01 |  |  |  |
|  |  | Weighted median | 1.043 (0.997, 1.090) | 6.50E-02 |  |  |  |
| Pulmonary embolism | Tryptophan | IVW-MRE | 1.021 (1.001, 1.043) | 4.39E-02 | 16 | 0.4719 | 0.4739 |
|  |  | MR Egger | 1.132 (0.860, 1.491) | 3.92E-01 |  |  |  |
|  |  | Weighted mode | 1.006 (0.952, 1.063) | 8.23E-01 |  |  |  |
|  |  | Weighted median | 1.013 (0.984, 1.042) | 3.81E-01 |  |  |  |
| Chronic heart failure | Tyrosine | IVW-MRE | 1.189 (1.051, 1.345) | 6.05E-03 | 3 | 0.361 | 0.3892 |
|  |  | MR Egger | 1.757 (1.013, 3.048) | 2.94E-01 |  |  |  |
|  |  | Weighted mode | 1.285 (1.102, 1.498) | 8.53E-02 |  |  |  |
|  |  | Weighted median | 1.164 (1.013, 1.337) | 3.26E-02 |  |  |  |
| Pulmonary embolism | Tyrosine | IVW-MRE | 1.002 (1.001, 1.004) | 8.67E-03 | 4 | 0.8143 | 0.5399 |
|  |  | MR Egger | 1.004 (0.999, 1.009) | 2.95E-01 |  |  |  |
|  |  | Weighted mode | 1.001 (0.997, 1.005) | 7.11E-01 |  |  |  |
|  |  | Weighted median | 1.002 (0.999, 1.005) | 1.88E-01 |  |  |  |
| Ischemic stroke | Valine | IVW-MRE | 1.070 (1.026, 1.115) | 1.59E-03 | 5 | 0.9486 | 0.5808 |
|  |  | MR Egger | 0.904 (0.524, 1.558) | 7.40E-01 |  |  |  |
|  |  | Weighted mode | 1.043 (0.916, 1.187) | 5.61E-01 |  |  |  |
|  |  | Weighted median | 1.039 (0.924, 1.167) | 5.27E-01 |  |  |  |

AA indicates amino acid; CVD, cardiovascular disease; CI, confidence interval; IVW-MRE, inverse variance weighted with multiple random effect; MR, Mendelian randomization; OR, odds ratio; SNP, single nucleotide polymorphism.

**Table S4. Causal relationships between AAs and CVDs with suggestive significance in EAS population**

| **Outcome** | **Exposure** | **Method** | **OR (95% CI)** | ***P*** | **No. of SNPs** | ***P_heterogeneity_*** | ***P_pleiotropy_*** |
| --- | --- | --- | --- | --- | --- | --- | --- |
| Coronary artery disease | Proline | IVW-MRE | 1.061 (1.020, 1.103) | 3.15E-03 | 4 | 0.3839 | 0.5374 |
|  |  | MR Egger | 1.080 (1.013, 1.152) | 1.43E-01 |  |  |  |
|  |  | Weighted mode | 1.069 (1.026, 1.115) | 5.16E-02 |  |  |  |
|  |  | Weighted median | 1.065 (1.023, 1.109) | 2.01E-03 |  |  |  |
| Angina pectoris | Glycine | IVW-MRE | 0.954 (0.918, 0.991) | 1.49E-02 | 10 | 0.2762 | 0.5616 |
|  |  | MR Egger | 0.941 (0.888, 0.998) | 7.61E-02 |  |  |  |
|  |  | Weighted mode | 0.954 (0.918, 0.992) | 4.10E-02 |  |  |  |
|  |  | Weighted median | 0.954 (0.917, 0.993) | 2.07E-02 |  |  |  |
| Chronic heart failure | Glycine | IVW-MRE | 0.950 (0.921, 0.981) | 1.48E-03 | 11 | 0.7274 | 0.6283 |
|  |  | MR Egger | 0.941 (0.891, 0.994) | 5.63E-02 |  |  |  |
|  |  | Weighted mode | 0.948 (0.906, 0.992) | 4.22E-02 |  |  |  |
|  |  | Weighted median | 0.948 (0.909, 0.989) | 1.24E-02 |  |  |  |
| Intracerebral hemorrhage | Histidine | IVW-MRE | 0.800 (0.696, 0.920) | 1.76E-03 | 7 | 0.8873 | 0.8761 |
|  |  | MR Egger | 0.761 (0.398, 1.455) | 4.46E-01 |  |  |  |
|  |  | Weighted mode | 0.850 (0.588, 1.229) | 4.20E-01 |  |  |  |
|  |  | Weighted median | 0.809 (0.614, 1.065) | 1.30E-01 |  |  |  |

AA indicates amino acid; CVD, cardiovascular disease; CI, confidence interval; EAS, East Asian; IVW-MRE, inverse variance weighted with multiple random effect; MR, Mendelian randomization; OR, odds ratio; SNP, single nucleotide polymorphism.

**References:**

1. Kettunen J, Demirkan A, Wurtz P*, et al.* Genome-wide study for circulating metabolites identifies 62 loci and reveals novel systemic effects of LPA. *Nat Commun*. 2016;7:11122.

2. Sakaue S, Kanai M, Tanigawa Y*, et al.* A cross-population atlas of genetic associations for 220 human phenotypes. *Nat Genet*. 2021;53:1415-24.

3. Shin SY, Fauman EB, Petersen AK*, et al.* An atlas of genetic influences on human blood metabolites. *Nat Genet*. 2014;46:543-50.

4. Liu X, Tong X, Zou Y*, et al.* Mendelian randomization analyses support causal relationships between blood metabolites and the gut microbiome. *Nat Genet*. 2022;54:52-61.

5. van der Harst P, Verweij N. Identification of 64 Novel Genetic Loci Provides an Expanded View on the Genetic Architecture of Coronary Artery Disease. *Circ Res*. 2018;122:433-43.

6. Koyama S, Ito K, Terao C*, et al.* Population-specific and trans-ancestry genome-wide analyses identify distinct and shared genetic risk loci for coronary artery disease. *Nat Genet*. 2020;52:1169-77.

7. Elsworth B., Lyon M., Alexander T.*, et al.* The MRC IEU OpenGWAS data infrastructure. *bioRxiv 20200810244293v1*. 2020.

8. Shah S, Henry A, Roselli C*, et al.* Genome-wide association and Mendelian randomisation analysis provide insights into the pathogenesis of heart failure. *Nat Commun*. 2020;11:163.

9. Malik R, Chauhan G, Traylor M*, et al.* Multiancestry genome-wide association study of 520,000 subjects identifies 32 loci associated with stroke and stroke subtypes. *Nat Genet*. 2018;50:524-37.
